# Supplementary material for: Theophylline: Old Drug in a New Light, Application in COVID-19 through Computational Studies
Source: Int J Mol Sci. 2022 Apr 9;23(8):4167. doi: 10.3390/ijms23084167 (PMC9030606; doi:10.3390/ijms23084167)
Supplement: Supplementary file 1 [file ijms-23-04167-s001.zip › ijms-1649567-supplementary.pdf]

International Journal of *Molecular Sciences*

MDPI

Supporting Materials

Theophylline: Old Drug in a New Light, Application in COVID-19 through Computational Studies

Luis M. Montaño [1], Bettina Sommer [2], Juan C. Gomez-Verjan [3], Genaro S. Morales-Paoli [3], Gema Lizbeth Ramírez-Salinas [4,5], Héctor Solís-Chagoyán [6], Zuly A. Sanchez-Florentino [6], Eduardo Calixto [7], Gloria E. Pérez-Figueroa [8,9], Rohan Carter [10], Ruth Jaimez-Melgoza [1], Bianca S. Romero-Martínez [1] and Edgar Flores-Soto [1,*]

[1] Departamento de Farmacología, Facultad de Medicina, Universidad Nacional Autónoma de México, CDMX, CP 04510, Mexico; lmmr@unam.mx (L.M.M.); jaimezruth@hotmail.com (R.J.-M.); biancasromero_@hotmail.com (B.S.R.-M.)

[2] Laboratorio de Hiperreactividad Bronquial, Instituto Nacional de Enfermedades Respiratorias "Ismael Cosío Villegas", CDMX, CP 14080, Mexico; bsommerc@hotmail.com

[3] Dirección de Investigación, Instituto Nacional de Geriatría, CDMX, CP 10200, Mexico; jver-jan@inger.gob.mx (J.C.G.-V.); mgenas.com@hotmail.com (G.S.M.-P.)

[4] Laboratorio de Diseño y Desarrollo de Nuevos Fármacos e Innovación Biotecnológica (Laboratory for the Design and Development of New Drugs and Biotechnological Innovation), Escuela Superior de Medicina, Instituto Politécnico Nacional, Plan de San Luis y Díaz Mirón S/N , Col. Santo Tomas, CDMX, CP 11340, Mexico; gemali86@hotmail.com

[5] Departamento de Inmunología, Instituto de Investigaciones Biomédicas, Universidad Nacional Autónoma de México, Circuito Escolar s/n, CP 4510, Mexico

[6] Laboratorio de Neurofarmacología, Instituto Nacional de Psiquiatría "Ramón de la Fuente Muñiz", CDMX, CP 14370, Mexico; hecsolch@imp.edu.mx (H.S.-C.); zulyarmandosf@gmail.com (Z.A.S.-F.)

[7] Departamento de Neurobiología, Dirección de Investigación en Neurociencias, Instituto Nacional de Psiquiatría "Ramón de la Fuente Muñiz", CDMX, CP 14370, Mexico; ecalixto@imp.edu.mx

[8] Unidad Periférica en el Estudio de la Neuroinflamación en Patologías Neurológicas, Instituto Nacional de Neurología y Neurocirugía. CDMX, CP. 06720, Mexico; gera.pfi3@gmail.com

[9] Laboratorio de Investigación en Inmunología y Proteómica, Hospital Infantil de México Federico Gómez, CDMX, CP. 06720, Mexico

[10] FRACGP/MBBS, Murchison Outreach Service Mount Magnet Western Australia, CP 6530, Australia; kingswood71@gmail.com

* Correspondence: edgarfs@comunidad.unam.mx; Tel.: +52-555-6232279

Methodology

Molecular modeling of the E protein from SARS-CoV-2

Based on the primary sequence YP_009724392 of the E protein from SARS-CoV-2, it was possible to build the E protein of this virus by employing the crystal structure PDB: 5X29, which corresponds to the E protein of SARS-CoV. The identity percentage between the sequence of the E protein of SARS-CoV-2 and the crystal structure is 91.379%. A three-dimensional model of the pentamer of the E protein of SARS-CoV-2 was built by using Modeller 10.1 Software [1]. Obtaining the three-dimensional structure of the Spike protein of SARS-CoV-2 Crystal 7WK3, downloaded from the pdb database [2], which is the Omicron variant SARS-CoV-2 Spike protein crystal; therefore, it is the crystal that we will use for the Docking.

Molecular docking

For the molecular docking of theophylline with both proteins (Spike and E of SARS-CoV-2) docking was performed directed at the RBD site (Spike protein) and the ion channel (E protein) respectively. The Vina program was used, with the following parameters: num_modes = 20, energy_range = 6 and exhaustiveness= 25. For the case of the Spike protein: center_x = 210.0, center_y = 170.0, center_z = 270.0, size_x = 60.00, size_y = 60.00 and

size_z = 60.00. And for protein E: center_x = -7.0,center_y = 1.0, center_z = -6.0, size_x = 35.00, size = 30.00 and size_z = 30.0 [3].

Table S1. Theophylline Simulation Conditions.

Parameter	Theophylline
**Lipophilicity (Log Units)**	0.89
**Binds To**	Albumin
**Fraction Unbound**	0.44
**Molecular Weight (g/mol)**	180.17
**Compound Type And Pka**	Acid / 8.8
**Solubilility At Ref-Ph (mg/L)**	14300
**Ref-pH**	6.5
**Metabolizing Enzymes**	CYP1A2
**Metabolizing Enzymes Clearence (1/Min)**	0.00843
**Renal Clearence**	0.15
**Administration Protocol**	Simple protocol
**Administration Type**	Intravenous bolus
**Dose (mg/Kg)**	1 and 10
**Dosing Interval**	Single

Table S2. Individuals Simulation Conditions.

	Healthy	***Renal impaired***
Population	Mexican American – White (NHAES, 1997)	Mexican American – White (NHAES, 1997)
Age (Years)	70	70
Gender	Male	Male
Weight (Kg)	74.43	74.43
Height (cm)	165.96	165.96
BMI (Kg/m²)	27.02	27.02
Body Surface Area (m²)	1.85	1.85
Hematocrit	0.46	0.33
EHC continuos fraction	0	1
GFR Specific (ml/min/100 g organ)	26.6	5
Metabolizing enzymes expression	CYP1A2	CYP1A2
